# The effect of narratives on perceived antibacterial resistance susceptibility: A randomized controlled experiment among Dutch medical students

**DOI:** 10.1177/13591053251400816

**Published:** 2026-01-12

**Authors:** Lieve Vonken, Math Candel, Gert-Jan de Bruijn, Kato Helsen, Stef Kremers, Francine Schneider

**Affiliations:** 1 Maastricht University, Netherlands; 2 University of Antwerp, Belgium

**Keywords:** perception, hospital personnel, health care, antibacterial resistance, randomized controlled trial

## Abstract

Healthcare professionals often perceive antibiotic resistance (ABR) as distant and abstract, thereby underestimating their susceptibility to it. This study examined whether reading or writing a narrative can enhance perceived susceptibility to ABR. In an experiment, Dutch medical students (*n* = 237) were randomly assigned to one of three conditions: reading or writing a narrative or reading non-narrative information (control). Perceived susceptibility was measured at three spatial distance levels: ‘you’, ‘your patients’, and patients abroad. Narratives, whether read or written, were not more effective than non-narrative information in increasing participants’ perceived susceptibility to ABR. Imaginability mediated the relationship between type of narrative and perceived susceptibility post-manipulation only at the distance level ‘your patients’. At this distance level, the pre-constructed narrative increased imaginability more than the self-constructed narrative. Future experimental studies should investigate the effect of adjusted narratives in increasing perceived susceptibility to ABR, a threat perceived as distant and abstract.

## Introduction

The WHO characterizes antibiotic resistance (ABR) as a top global public health threat (World Health Organization, 2024). ABR occurs when changes in bacteria make them less susceptible to antibiotics, which complicates infection treatment and increases the risk of worse patient outcomes and disease spread (World Health Organization, 2024). Major causes and consequences of ABR exist in healthcare, making healthcare professionals key in curbing ABR (Malani et al., 2024). To curb ABR, healthcare professionals are primarily recommended to adhere to infection prevention measures, such as hand hygiene, and to prescribe antibiotics rationally, evidence-based, and preferably after diagnosis (Wall, 2019; World Health Organization, 2015). This is highlighted in antibiotic stewardship programs and infection prevention and control initiatives. Despite the positive effects of stewardship initiatives on antibiotic use (Zay Ya et al., 2023), antibiotic-resistant infections occur increasingly often (de Greeff et al., 2024).

As ABR spreads, it becomes increasingly crucial for healthcare professionals to act against it. This highlights the importance of understanding how to motivate healthcare professionals’ preventive actions. One explanation for their limited action against ABR is that they recognize the seriousness of ABR but question their own susceptibility (Krockow et al., 2019; Vonken et al., 2024, 2025). This is in line with the Extended Parallel Process Model (Witte, 1994), which suggests that someone’s intention to protect themselves against risk is largely determined by the risk appraisal (severity and susceptibility) and coping appraisal (response efficacy and self-efficacy). Empirical evidence confirms that both high threat perception and high perceived efficacy are needed to promote action against a threat (Peters et al., 2013). Given that healthcare professionals often question their susceptibility to ABR, increasing their perceived susceptibility could be a first step to encouraging their action against ABR.

Risk communication can be applied to support sufficiently high perceived susceptibility. However, two of ABR’s characteristics impede communication about ABR in traditional quantitative scenarios. Firstly, the complexity of ABR hampers defining the problem exhaustively and expressing its burden quantitatively (Chandler, 2019). ABR is a collective noun for multiple complex biological processes that all reduce the effectiveness of antibiotics. Moreover, resistant bacteria spread freely across nature, animals, and humans (World Health Organization, 2023). The interconnectedness of ABR’s causes and consequences does not allow the identification of singular behaviors that lead to distinct ABR consequences (Chandler, 2019). Probalistic quantitative risk scenarios often cannot grasp such difficult-to-predict problems (Butler et al., 2020). As a result, current risk communication about ABR generally fails to accurately portray its risks. ABR and its consequences are oversimplified, for example, newspapers, describe ‘superinfection’ instead of the causal process of ABR (Capurro, 2020). For healthcare professionals, the consequences of ABR are especially complex because they can affect patients abroad, their patients, and themselves. Secondly, the consequences of ABR occur in the future, not directly (Roope et al., 2019). Even if the future consequences of a problem can be portrayed quantitatively, insights from economics suggest that the evaluation of future events is often discounted (i.e. delay discounting). This means that the longer it takes for consequences to occur, the less relevant they are perceived (Frederick et al., 2002; Roope et al., 2019). Nevertheless, an alternative perspective is that, from the standpoint of countries with low ABR rates, the consequences of ABR are already visible in other countries (Vonken et al., 2024). Although these consequences of ABR occur in the present (small temporal distance), they take place in other countries, which creates a large spatial distance.

To overcome the challenges of traditional quantitative ABR risk communication, narratives have been recommended (McCall et al., 2023). Narratives are stories that connect actions and events (Cho and Friley, 2014). They may be particularly effective for communicating about ABR, a distant and abstract problem, because they have proven effective in risk communication by bridging a spatial or temporal gap between the present and a temporally and/or spatially distant consequence (de Graaf et al., 2016; Zebregs et al., 2015). Compared to different types of quantitative communication, they are less likely to prompt counterarguing and are therefore more likely to foster risk acceptance (Cho and Friley, 2014; Moyer-Gusé, 2008). In addition to demonstrating the ability to change beliefs, attitudes, intentions, and behaviors (Braddock and Dillard, 2016), narratives were found to be more effective in reducing ABR misconceptions than factual explanations (Pickett et al., 2022).

Narratives are most effective when they allow for transportation, feeling engaged, or being ‘lost in the story’ (Green and Brock, 2000; Hinyard and Kreuter, 2007; van Laer et al., 2014). For example, in an experiment with narratives about skin cancer, greater transportation was associated with stronger gut feelings of risk and stronger intentions to perform protective behavior (i.e. skin self-examination and looking for information; Dillard et al., 2018). Transportation is closely linked to imaginability (van Laer et al., 2014). To achieve transportation in a narrative, it is recommended that someone writes the narrative themselves. This should ensure that the situation described is imaginable (Eldredge et al., 2016; Mrkva et al., 2018). An experimental study achieved more imaginability and higher perceived susceptibility to Chlamydia when participants wrote their own risk scenario, describing how they contracted Chlamydia, compared to when they read a predefined scenario (Mevissen et al., 2012).

It is unknown whether imagining and writing about a complex and abstract problem such as ABR has the same effect. For example, empirical research shows that to promote an action whose result is delayed instead of immediate, a more distant framing of this situation is most effective. Distant and thus abstract thinking in this case leads to stronger self-control (Fujita and Roberts, 2010; Zwickle and Wilson, 2013). In line with this, a study investigating the effect of distance-framed narratives about ocean plastic pollution found that distance-framing was more persuasive than spatially close framing (Liu and Yang, 2023).

### Hypotheses

Promoting perceived personal susceptibility to ABR among healthcare professionals is vital. Narratives can effectively enhance perceived susceptibility, especially when individuals create the stories themselves, making them more imaginable and personally relevant (Eldredge et al., 2016; Mevissen et al., 2012). This study applies an experimental design to investigate the effect of reading or writing a narrative on perceived susceptibility to ABR for themselves, their patients, and patients abroad. Susceptibility before and after the experiment is measured, and the type of risk information is manipulated (pre-constructed narrative, self-constructed narrative, or control [non-narrative information]). Because imaginability is a known mediator of the effect of narratives, the possible mediating effect of imaginability is also investigated.

The primary hypothesis is that the perceived susceptibility to ABR post-manipulation will be: a) higher for participants in the experimental conditions (reading or writing a narrative) than for participants in the control condition (reading non-narrative information); and b) higher for participants in the writing than for participants in the reading condition. Susceptibility is measured at multiple levels of spatial distance: for themselves, their patients, and patients abroad.

The secondary hypothesis is that the effect described under primary hypothesis b is mediated by imaginability. Specifically, it is hypothesized that the imaginability of the narrative will be higher for participants in the writing condition than for participants in the reading condition, and that the perceived susceptibility to ABR will be higher for participants who rate the imaginability of the narrative as higher.

## Methods

This study applied a three-group experimental design. Participants were randomly allocated to one of three conditions. In these conditions, participants wrote their own risk scenario, read a pre-constructed risk scenario, or read non-narrative information (control condition). The allocation ratio was 1:1:1. In all conditions, the scenario or information was presented as a screenshot of a WhatsApp chat. WhatsApp is the most used social media in the Netherlands and is therefore a realistic means of communication for the participants (van der Veer et al., 2025). Baseline data (background variables and risk perception, including perceived susceptibility) and post-manipulation data (evaluation of the condition and risk perception, including perceived susceptibility) were collected. The differences in susceptibility between the three groups post-manipulation were examined, correcting for baseline differences in susceptibility and awareness. Moreover, a mediating effect of imaginability was investigated. Ethical approval for this study was granted by the Maastricht University Faculty of Health, Medicine and Life Sciences Research Ethics Committee (FHML-REC/2024/038). The analysis plan was registered on the Open Science Framework (OSF; osf.io/5dz69).

### Participants

Participants were medical students doing internships in the Master of Medicine (Dutch track) at Maastricht University. Students were chosen as our target group because they most recently received education on ABR, thus providing insights on the current state of education. Only students in the internship stage of their study were selected to ensure that they had sufficient clinical experience. To ensure a representative sample, all students doing these internships were eligible for participation. Sample size calculations were carried out to determine the intended sample size. The required sample size for the primary hypotheses was calculated in G*power (Faul et al., 2007). Based on the effect sizes found by (Mevissen et al., 2012). Mevissen et al. (2012), that is, η_p^2 = 0.05 on perceived susceptibility and η_p^2 = 0.09 on threat perception, we also assume a medium effect size for this study (*d* = 0.5). With 80% power for a two-sided test and a Bonferroni adjusted type I error rate alpha* = 0.01667 (=0.05/3, accounting for three tests on susceptibility at different distance levels), the required sample size is *N* = 86 per treatment group. Since the pretest measure will be included in the analysis as a covariate (assuming a correlation of 0.6 between pretest and posttest), the total minimum sample size across all groups can be reduced to 166 participants. To ensure equal allocation across the three treatment groups, the final sample size was set to 168 participants. For the sample size calculation for the secondary hypothesis, we refer to the preregistration of this study.

### Procedure

From August 20, 2024, to March 6, 2025, participants were recruited on teaching days which are scheduled once every 2–4 weeks. Specifically, students were recruited (a) in the surgery internship, at the start of the microbiology workshop; (b) at the family medicine teaching day, at the end of the microbiology workshop; (c) at the start of the research internship, at the start or end of a qualitative research workshop.

#### Invitation to participate

LV or thoroughly instructed colleagues asked students to participate through a short explanation of the study (based on https://osf.io/dkty3/). Participants followed a link or QR code to Qualtrics, where they read the information letter and actively indicated informed consent before starting the questionnaire.

#### Questionnaire

The questionnaire consisted of three parts (https://osf.io/k6n32/):

- Part 1 – baseline: background information, risk perception of ABR, and response- and self-efficacy of acting against ABR;
- Part 2 – manipulation: three conditions, all consisting of a screenshot of a WhatsApp chat;
- Part 3 – post-manipulation: evaluation of the intervention, and risk perception of ABR.

After filling in part 1, participants were randomly allocated to one of the three conditions by Qualtrics, making the experiment double-blind. Qualtrics ensured groups that are equal in size and comparable in participant characteristics. After every recruitment day, LV checked if any participants needed to be excluded from the analysis and adjusted the Qualtrics counts of participants in each condition accordingly. At the end of the questionnaire, participants could indicate if their anonymous data from this project could be published on OSF (form based on https://osf.io/vt4fz/).

#### Manipulation

**Figure 1.**
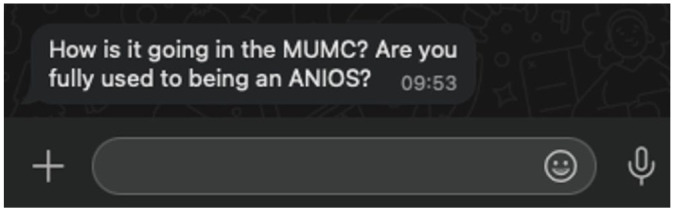
A screenshot of the WhatsApp message in the writing condition. ANIOS stands for ‘arts niet in opleiding tot specialist’, describing a medical doctor not in specialty training, often similar to interns in the United States.

The non-narrative information text described what ABR is. In the narrative writing condition, the participant is instructed to write a realistic response, describing how they did something that worsened ABR, as a response to a message from another student (Figure 1). In the narrative reading condition, another student describes how they inappropriately prescribed broad-spectrum antibiotics and are then confronted with how severe ABR can be when seeing another patient.

### Outcomes

At baseline the questionnaire inquired about the stage of education (year of study and completion of which internships), pre-existing awareness (two items; awareness of causes and consequences of ABR in the hospital, Cronbach’s alpha 0.74) of and experience with ABR (three items; inquiring about experience in their personal life, education, and work). A modified version of the Risk Behavior Diagnosis scale measured perceived susceptibility and perceived severity at baseline and post-manipulation (Witte et al., 1996). The primary outcome, perceived susceptibility, was measured at three distance levels: for patients abroad, your patients, and ‘you’ (respective Cronbach’s alphas at baseline: 0.64; 0.76; 0.75 and post-manipulation: 0.79; 0.84; 0.77). Post-manipulation, the intervention was evaluated by inquiring about imaginability (four items, Cronbach’s alpha 0.74) and the relation between cause and effect (one item). The Cronbach’s alpha for imaginability increased (0.76) when the item about likelihood of experience was removed and increased even further when the item about being able to vividly imagine the situation was removed (0.81). Because being able to vividly imagine the situation was the main measure of imaginability (see preregistration), with likelihood of experience as the expected most similar variable, the index was deemed unreliable and only the score given for being able to vividly imagine the situation was used in the analyses.

### Data preparation

LV anonymized the questionnaire data before it was made accessible to the research team. Data was analyzed in SPSS and Microsoft Excel, using UM licenses. The condition (writing narrative, reading narrative, non-narrative information) was dummy-coded into three dummies. We only included complete cases. We also excluded any questionnaires that were not completed during a recruitment moment (i.e. with a deviant start date). After all students in a recruitment group completed the questionnaire, they were informed of the hypotheses of this study. For any questionnaires started later, participants were likely not invited by the researchers (i.e. the link was forwarded by a participant) and/or knew the hypotheses of this study, which may have biased their answers. All narratives describing a participant’s action in a hospital that leads to ABR were included.

### Analysis

Frequencies (%), means (*M*), and standard deviations (SD) were reported descriptively. Baseline differences between the three conditions were analyzed using the Chi-square test for dichotomous and categorical variables (year of internship and experience) and a one-way ANOVA for the continuous variables (awareness and perceived susceptibility). Baseline differences in perceived susceptibility between the three distance levels were analyzed using a one-way repeated measures ANOVA with Greenhouse and Geisser correction and pairwise comparisons with Bonferroni correction for multiple testing. Differences between the evaluation of the manipulation between the conditions were analyzed using a one-way ANOVA (vividly imagining sending and receiving the message, vividly imagining the situation, the likelihood of experiencing the situation, and the perceived presence of a relationship between cause and effects). When Levene’s test indicated heterogeneity of variances, a Welch ANOVA was used. When comparing three conditions and when a Welch ANOVA indicated significant results, Games-Howell corrected pairwise comparisons were applied.

The primary hypothesis was tested with a separate linear regression for each of the three distance levels of susceptibility. The differences between the three groups on post-manipulation susceptibility were examined, correcting for baseline susceptibility and awareness. This analysis was run twice to include all three condition dummies. For the secondary hypothesis, participants in the control condition were excluded from the analysis. The joint significance test was applied three times, once for each distance level. Firstly, the differences between the two groups in the imaginability score were examined, correcting for baseline susceptibility and awareness. Secondly, the effect of imaginability on post-manipulation susceptibility was examined, correcting for baseline susceptibility, awareness, and the condition (writing vs reading). Mediation was confirmed only if the condition was significantly related to imaginability in the first model, and if imaginability contributed significantly to susceptibility in the second model. We used the standard *p* < 0.05 criterion in a two-sided test to determine whether the linear regression analysis suggests that the results differ significantly from those expected if the null hypothesis were correct. A Bonferroni correction for multiple testing was applied when testing the primary and secondary hypotheses.

As a manipulation check, the length and content of the narratives written by the participants were analyzed using content analysis. After pilot coding and discussing 15 narratives, the remaining narratives were double-coded by LV and KH in Microsoft Excel (kappa 0.91). Disagreements were discussed and resolved, resulting in 100% agreement. Themes were created deductively, based on the requirements for the narrative as described in the questionnaire: respondents performing an action (i), which can be considered a mistake (ii), which results in increased ABR (iii). Additional themes were added inductively during pilot coding, based on the content of the narratives.

## Results

**Figure 2.**
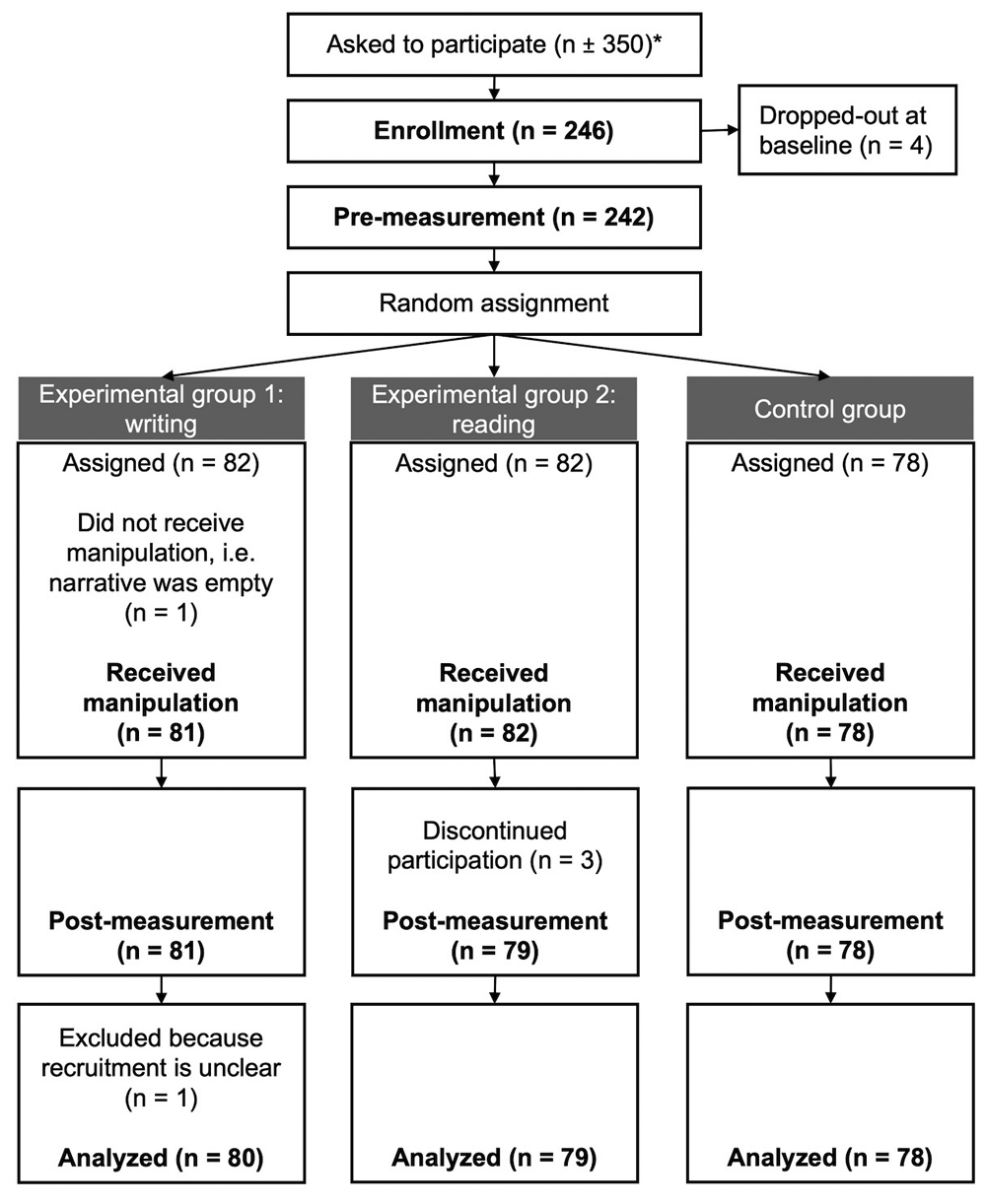
Recruitment flowchart. *Estimate based on about 1 per 20 medical students not participating in the surgery group and about 50 medical students not participating in the other groups. The latter number is higher because students were sometimes recruited at the end of the teaching day instead of at the beginning, during teaching hours.

In total, 246 participants enrolled, and data from 237 participants were analyzed (Figure 2). Participants whose data were analyzed were recruited during their surgery internship (*n* = 170), during their family medicine teaching internship (*n* = 37) and their research internship (*n* = 30).

### Descriptives for sample and baseline comparisons

Most participants were in their first internship year (66.7%, *n* = 158) and had experience with ABR in their work or education (72.3%, *n* = 194). Only 7.2% (*n* = 17) of participants gave an example of experience with ABR in their personal life. Chi-square tests showed no statistically significant association between condition and year of internship, or condition and experience with ABR in personal life, education, or work (see Appendix).

On average, participants indicated relatively high awareness of causes and consequences of ABR in the hospital, combined *M* ± SD 5.21 ± 1.08 on a scale of 1–7, where 7 indicates very high awareness. Awareness was not statistically significantly different between conditions (see Appendix).

**Figure 3.**
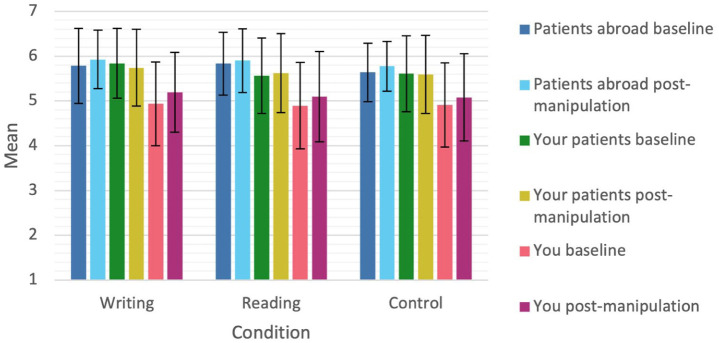
Susceptibility for different distance levels, at baseline and post-manipulation, per condition. *M* ± SD.

Perceived susceptibility per condition group is displayed in Figure 3. At baseline, participants perceived the susceptibility to ABR as higher for patients outside the Netherlands (M ± SD: 5.75 ± 0.74) than their own patients (5.67 ± 0.83) than themselves (4.91 ± 0.94). Susceptibility was not statistically significantly different between conditions at any distance level (see Appendix). The difference in perceived susceptibility for the different distance levels was statistically significant, between patients abroad and ‘you’ (respectively *M* = 5.75 and *M* = 4.91, mean difference 0.84, 95% CI 0.66 to 1.01, *p* < 0.001) and ‘your patients’ and ‘you’ (respectively *M* = 5.67 and *M* = 4.91, mean difference 0.76, 95% CI 0.63–0.88, *p* < 0.001).

### Manipulation check: narratives

An example narrative is shown below. The mean word count was 66.0 ± 33.1. Content analysis showed that most participants (95.0%, *n* = 76) performed the action in the narratives themselves (Table 1). Despite the instruction to describe a mistake, some participants (7.2%, *n* = 6) described a neutral action (e.g. following instructions from a supervisor). The most often mentioned actions were irrational prescribing (53.8%, *n* = 43) or not consulting cultures or other existing information (35.0%, *n* = 28). The most mentioned consequences were increased ABR (51.3%, *n* = 41), consequences for the patient (38.8%, *n* = 31), or medical consequences (26.3%, *n* = 21). Negative affect was described in half of the narratives (51.3%, *n* = 41).

Example narrative (Translated with DeepL.com, free version): “I didn’t have such a good day today, I think I made a major mistake. . . So a patient was admitted with urosepsis and I was maybe a little too enthusiastic and I immediately started administering ceftriaxone and I didn’t take blood cultures beforehand. The patient had a high fever so according to the protocol you should always take cultures. Because I didn’t take cultures I may not be able to find out exactly what bacteria it is and I can’t switch to a narrow spectrum antibiotic.”

**Table 1.** Results of the content analysis of the narratives. Occurring themes with counts of occurrence.

Themes		Count	Percentage
Actions	The respondent performs the action themselves	76	95.0
	The respondent does not make a mistake	6	7.2
*Type of action*	*Irrational prescribing*	43	53.8
	*Not consulting cultures or other existent information*	28	35.0
	*Insufficient hygiene*	1	1.3
	*Insufficient instructions for the patient*	1	1.3
	*Unclear*	7	8.8
Consequences	Increased ABR	41	51.3
	For the patient (often mild, e.g., slight pain)	31	38.8
	Medical consequences (e.g., treatment complication)	21	26.3
	For you (excluding emotional consequences)	3	3.8
	Other	5	6.3
	*Negative affect*	41	51.3
	*No consequences mentioned*	11	13.8

Percentages are calculated as fraction of the total number of narratives (*n* = 80). No themes are mutually exclusive. Italics is to indicate a ‘sub-category’.

### Manipulation check: participant evaluation

**Figure 4.**
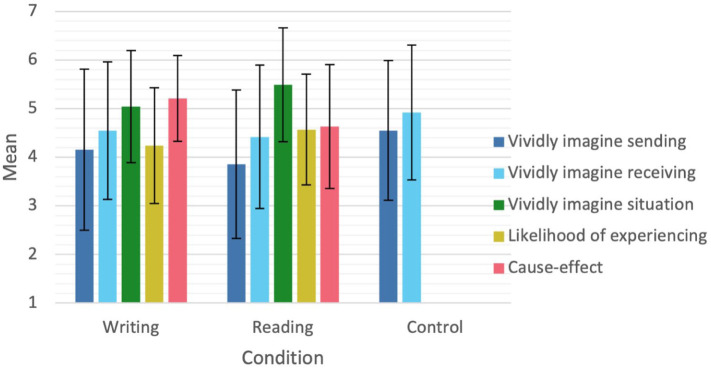
The participants’ evaluation of the manipulation. *M* ± SD.

Participants of all conditions could vividly imagine sending and receiving the message (mean per condition ⩾ 3.5 out of 7, where 7 indicated very high ease of imagination, Figure 4). In the experimental conditions, participants could vividly imagine the situation and rated the likelihood of experiencing the situation and the perceived presence of a relationship between cause and effect relatively high (mean per condition ⩾ 4 out of 7, where7 indicated strong agreement with the statement).

Vividly imagining receiving the message and the likelihood of experiencing were not statistically significantly different between conditions (see Appendix). Vividly imagining sending the message was statistically significantly different between the three conditions (Welch’s *F*(2, 155.73) = 4.280, *p* = 0.02), and post hoc tests showed that reading and control differed significantly (respectively *M* = 3.86 and *M* = 4.55, mean difference −0.69, *p* = 0.01). Vividly imagining the situation and the cause-and-effect relationship were statistically significantly different between the conditions reading and writing, respectively *F*(1,157) = 6.11, *p* = 0.02 with *M* = 5.49 and *M* = 5.04 and Welch’s *F*(1, 138.68) = 11.12, *p* = 0.001 with *M* = 4.63 and *M* = 5.21. In the writing condition, participants had more difficulty imagining the situation, but more clearly recognized a cause-and-effect relationship.

### Effect analyzes

Multiple regressions showed no significant association between the condition and perceived susceptibility post-manipulation at any of the distance levels (Table 2). The model included perceived susceptibility at baseline, the dummy variables of the experimental conditions, and awareness. Only perceived susceptibility at baseline added statistically significantly to the model (*p* < 0.001 for patients abroad, *p* < 0.001 for ‘your patients’, *p* < 0.001 for ‘you’).

**Table 2.** Multiple regression results of the effect on susceptibility.

Analyses	Patients abroad	‘Your patients’	‘You’
Model	*F*(4, 232) = 38.29, *p* < 0.001, *R*^2^ = 0.40	*F*(4, 232) = 45.97, *p* < 0.001, *R*^2^ = 0.44	*F*(4, 232) = 95.92, *p* < 0.001, *R*^2^ = 0.62
Writing versus control	β = 0.05, *p* = 0.37	β = −0.01, *p* = 0.88	β = 0.04, *p* = 0.35
Reading versus control	β = 0.02, *p* = 0.81	β = 0.03, *p* = 0.57	β = 0.01, *p* = 0.77
Reading versus writing	β = −0.04, *p* = 0.51	β = 0.04, *p* = 0.47	β = −0.03, *p* = 0.52

The results of the model, and of the contribution of the condition to the model.

### Mediation analysis

The joint significant test only showed a significant association between the condition and imaginability (first part) and between imaginability and perceived susceptibility post-manipulation (second part) at the distance level ‘your patients’ (Table 3). This means that imaginability is only a mediator of a relationship between the condition and perceived susceptibility post-manipulation at the distance level ‘your patients’. In contrast to the hypothesis, reading led to higher imaginability than writing a narrative at all distance levels. In line with the hypothesis, higher imaginability led to higher perceived susceptibility for ‘your patients’ post-manipulation.

**Table 3.** Multiple regression results of the mediation analyses.

Analyses	Patients abroad	‘Your patients’	‘You’
Joint significance test first part, imaginability as dependent variable			
Model	*F*(3, 155) = 2.72, *p* = 0.05, *R*^2^ = 0.05	F(3, 155) = 2.73, *p* = 0.05, *R*^2^ = 0.05	*F*(3, 155) = 2.71, *p* = 0.05, *R*^2^ = 0.05
Writing versus reading	β = −0.20, *p* = 0.013	β = −0.20, *p* = 0.011	β = −0.20, *p* = 0.013
Joint significance test second part, susceptibility as dependent variable			
Model	*F*(4, 154) = 25.15, *p* < 0.001, *R*^2^ = 0.40	*F*(4, 154) = 31.90, *p* < 0.001, *R*^2^ = 0.45	*F*(4, 154) = 63.03, *p* < 0.001, *R*^2^ = 0.62
Imaginability	β = 0.10, *p* = 0.108	β = 0.21, *p* = 0.001	β = 0.10, *p* = 0.06

The results of the models, and of the contribution of the condition (part one) and imaginability (part two) to the model. Bonferroni adjusted alpha = 0.017.

## Discussion

This study applied a three-group experimental design to investigate the effect of narrative risk communication on medical students’ perceived susceptibility to ABR. Narratives were not more effective than non-narrative risk information in increasing participants’ susceptibility to ABR, nor was writing a narrative more effective than reading a narrative. Only at the distance level ‘your patients’ was this effect mediated by imaginability. This study is among the first in the ABR field investigating the effects of one type of risk communication. Experimentally testing different types of risk communication for ABR is the only way to find an effective means of communication.

An explanation for both narratives not being more effective than the control might be that neither of the experimental conditions contained an obvious relationship between cause and effect, while also promoting imaginability. In the self-constructed narratives, the relationship between cause and effect was more easily recognized, while the pre-constructed narratives promoted imaginability more. This is relevant because for scenario-based risk information, a ‘plausible scenario with a cause and an outcome; imagery’ is a parameter for use (Kok et al., 2016; Mevissen et al., 2012). Especially when describing future events such as ABR, narratives with detailed episodic descriptions are suggested to create vivid mental images and counteract optimism bias, that is, overestimating the chance of a positive outcome (Richter et al., 2023).

That imaginability was not sufficiently provoked is also a potential explanation for why writing a narrative did not increase susceptibility to ABR more effectively than reading a narrative. In contrast to the hypothesis, reading led to higher imaginability than writing a narrative at all distance levels. Higher imaginability was hypothesized to lead to a higher increase in perceived susceptibility (Dillard et al., 2018). Studies in which participants’ imaginability is increased successfully by having them write about a risky situation and its negative result most often study very personal actions and consequences, such as contracting Chlamydia (Mevissen et al., 2012). Consequences of ABR might be too complex to imagine. Although participants had some clinical experience in their internships, they might not have had sufficient clinical experience to imagine the consequences of an action on ABR. Something that healthcare professionals with more experience might be able to do more easily.

Despite the experimental conditions not being more effective in increasing perceived susceptibility post-manipulation than the control, what stands out is that at the distance level ‘your patients’, imaginability was a mediator of the relationship between the condition and perceived susceptibility post-manipulation. A possible explanation for this is that another mediator, which was not included in the analyses, cancels out the effect of the narratives on perceived susceptibility post-manipulation mediated by imaginability. Possible additional mediators are the constructs measured as an evaluation of the manipulation. Preliminary analysis does not suggest cause-and-effect relationship is a mediator (not published). Further research is needed to fully understand the relationship between narratives, possible mediators, and perceived susceptibility. Other constructs that may have influenced the effectiveness of the narrative include emotion and gain or loss framing (Hameleers, 2021; Richter et al., 2023).

An explanation for this mediation effect only being found at the distance level ‘your patients’ is that both narratives might have emphasized the consequences for the patient too strongly. The self-constructed narratives often mentioned medical consequences and consequences for the patient. Consequences for the participant themselves were rarely mentioned. This might have only triggered thinking about ‘your patients’ and therefore could only potentially change susceptibility at that level. That susceptibility to ABR is indeed perceived differently at different distance levels is shown by the descriptive statistics. At baseline, participants perceived the susceptibility to ABR to be highest for patients abroad, followed by their patients, and finally themselves. It is suggested that to promote an action of which the result is delayed instead of immediate, more distant framing of this situation is most effective because this leads to abstract thinking which in this case leads to stronger self-control (Fujita and Roberts, 2010; Zwickle and Wilson, 2013). Either a narrative that highlights consequences for ‘you’ or a narrative that highlights distant and abstract consequences might have been more effective.

What further stands out is that while imaginability was evoked more in the reading than in the writing condition, the cause-and-effect relationship was more visible in the writing than in the reading condition. That the pre-constructed scenario did not sufficiently contain a cause-and-effect relationship could indicate a deficiency in this specific scenario. Still, in neither condition were the narratives rated as extremely imaginable nor was the cause-and-effect relationship extremely obvious. This points to the difficulty of constructing an imaginable narrative in which an action of an individual leads to worsened ABR.

Possibly, the cause-and-effect relationship being more obvious in the self-constructed narratives was associated with these narratives being less imaginable. In other words, participants wrote stories with a clear cause-and-effect relationship to comply with the assignment, however, they may have rated these stories as less imaginable because they struggled to imagine their own actions resulting in increased ABR. This idea is supported by the content of the self-constructed narratives, which often includes other consequences than increased ABR. While half of the self-constructed narratives explicitly mentioned increased ABR – as requested in the assignment – many described only consequences for the patient (often mild) and medical consequences. Some narratives describe no consequences related to ABR at all. Participants listing other consequences than increased ABR might be a result of the consequences of ABR currently being too abstract and distant for participants to imagine to be a result of their own actions. Indeed, Dutch healthcare professionals often perceive causes of ABR to be external and consequences to be abstract (Vonken et al., 2025). This problem is part of the reality of ABR in the Netherlands, where currently one healthcare professionals’ action rarely leads to a direct increase in ABR, let alone to strongly more adverse patient outcomes (Roope et al., 2019). It has been suggested that the consequences of ABR for patients feel more psychologically distant because even patients infected with resistant bacteria can often still be treated with newer antibiotics or combination therapy, limiting the perceived consequences of ABR (Harring and Krockow, 2021).

The main limitation of this study is that the instructions given might not have sufficiently promoted the imaginability of the situation. What supports this is that participants in the control group could more easily imagine sending the message vividly than participants in the experimental conditions. The instructions are highly relevant for the outcome of a writing task (Nazarian and Smyth, 2013), and previous experiments showed that writing a narrative can increase simulation fluency more than reading a narrative (Mrkva et al., 2018). However, an alternative explanation for the low imaginability of the self-constructed narratives is that the consequences of ABR were too difficult to imagine for our participants. Specifically, the medical students might not have had sufficient clinical experience to imagine the consequences of ABR. A further limitation of this study is the use of a single-item measure for imaginability. Single-item measures are less reliable and might thus induce attenuation of, in this case, the mediation effect. However, as single-item measures can be used with relatively homogeneous constructs (Loo, 2002), the current findings are still valuable.

A strength of this study is that we included a homogeneous participant group randomly allocated to three conditions, one of which was a control group. This allowed studying the variable of interest with as little extraneous variables as possible influencing the results. Additional strengths are the pre-designed protocol, which improved methodological rigor and enhanced transparency, and the pre-determined sample size, which ensured that the methods and findings answered the research question with sufficient power. This contributes to the reliability and credibility of the findings.

Seeing the considerations and limitations mentioned, future research is needed to elucidate the effect of narratives on perceived ABR susceptibility. On the one hand, further experimental studies are needed to test to what extent students and healthcare professionals can imagine the consequences of ABR. On the other hand, narratives that describe these consequences in an imaginable manner need to be developed. Further, experimental studies should test whether different narratives have different effects. Specifically, at what distance level, with which cause, and which outcome narratives are most effective. A narrative that is either closer or more distant might achieve a stronger increase in susceptibility at those distance levels. In addition to the distance levels studied here, consequences for ‘this one patient’ might be perceived differently. Psychological processes, such as emotions, heuristics, and cognitive biases, should be considered in this (Richter et al., 2023). In addition, these studies should apply a more sensitive measure of susceptibility to ensure even a slight increase is detected. Lastly, these studies should aim to include healthcare professionals with even more clinical experience than students. ABR risk communication by use of narratives has the most potential for use in education, for example, in antibiotic stewardship. Future research is needed to include additional constructs (such as efficacy) that predict and ultimately influence behavior.

## Conclusion

Reading or writing narratives about increasing ABR is not effective in enhancing perceived susceptibility among Dutch medical students. Neither reading nor writing a narrative simultaneously promoted imaginability and contained a clear cause-and-effect structure, according to the participants. Future experimental studies should investigate the effect of adjusted narratives or narrative writing instructions in increasing perceived susceptibility to a problem like ABR that is perceived as distant and abstract.

## Appendix

### Descriptives and baseline statistics

#### Year of internship

First year: 66.7%, *n* = 158; second year: 13.5%, *n* = 32; third year: 8.9%, *n* = 21; fourth year or later: 11.0%, *n* = 26

**Table:**

Condition		Frequency	Percent
Writing	1	55	68.8
	2	11	13.8
	3	8	10.0
	4 or later	6	7.5
	Total	80	100.0
Narrative	1	50	63.3
	2	11	13.9
	3	5	6.3
	4 or later	13	16.5
	Total	79	100.0
Control	1	53	67.9
	2	10	12.8
	3	8	10.3
	4 or later	7	9.0
	Total	78	100.0

#### Internship completed (optional question)

**Table:**

	Writing (*n*)	Reading (*n*)	Control (*n*)
Neuroscience	17	13	15
Surgery	26	18	20
Internal medicine	48	43	43
Woman, mother, child	17	13	15
Family medicine	5	2	6
Participation in healthcare	5	1	3
Research participation	16	14	12
Internship of choice	18	18	22

#### Current internship (optional question)

**Table:**

Condition			Frequency	Percent
Writing	Valid	Surgery	52	65
		Family medicine	12	15
		Research participation	10	12.5
		Total	74	92.5
	Missing	System	6	7.5
Reading	Valid	Surgery	55	69.6
		Family medicine	10	12.7
		Research participation	13	16.5
		Total	78	98.7
	Missing	System	1	1.3
Control	Valid	Surgery	56	71.8
		Family medicine	8	10.3
		Research participation	12	15.4
		Internship of choice	1	1.3
		Total	77	98.7
	Missing	System	1	1.3

#### Awareness and experience

**Table:**

Condition			Awareness	ABR experience			Aware of consequences	Aware of causes
				In personal life	In education	At work		
Writing	*N*	Valid	80	80	80	80	80	80
		Missing	0	0	0	0	0	0
	Mean		5.2125	0.1	0.83	0.38	5.34	5.09
	Std. error of mean		0.12123	0.034	0.043	0.054	0.132	0.139
	Std. deviation		1.08434	0.302	0.382	0.487	1.179	1.245
	Variance		1.176	0.091	0.146	0.237	1.391	1.549
	Minimum		2	0	0	0	1	2
	Maximum		7	1	1	1	7	7
	Percentiles	25	5	0	1	0	5	5
		50	5.5	0	1	0	6	5
		75	6	0	1	1	6	6
Reading	N	Valid	79	79	79	79	79	79
		Missing	0	0	0	0	0	0
	Mean		5.2342	0.09	0.7	0.39	5.34	5.13
	Std. error of mean		0.10065	0.032	0.052	0.055	0.111	0.12
	Std. deviation		0.89455	0.286	0.463	0.491	0.986	1.067
	Variance		0.8	0.082	0.214	0.241	0.971	1.138
	Minimum		3	0	0	0	3	2
	Maximum		7	1	1	1	7	7
	Percentiles	25	5	0	0	0	5	5
		50	5.5	0	1	0	6	5
		75	6	0	1	1	6	6
Control	N	Valid	78	78	78	78	78	78
		Missing	0	0	0	0	0	0
	Mean		5.0577	0.06	0.77	0.31	5.23	4.88
	Std. error of mean		0.10362	0.028	0.048	0.053	0.112	0.117
	Std. deviation		0.91517	0.247	0.424	0.465	0.992	1.032
	Variance		0.838	0.061	0.18	0.216	0.985	1.064
	Minimum		2.5	0	0	0	3	2
	Maximum		6	1	1	1	7	6
	Percentiles	25	5	0	1	0	5	5
		50	5.5	0	1	0	5	5
		75	5.5	0	1	1	6	6

ABR experience in work or education included lectures, practicals, assignments, and patients with resistant infections, sometimes at their side jobs.

Twelve of the participants who indicated to have experience with ABR in their personal life stated that they or their family members had a resistant infection, two described it as a problem in a country they visited (or were born), three described discussing ABR in their personal life.

#### Baseline comparisons

##### Year of internship and experience per condition

Chi-square tests for association showed no statistically significant association between condition and year of internship, χ^2^(6) = 4.44, *p* = 0.69, or condition and experience with ABR in personal life, education or work, respectively χ^2^(2) = 0.69, *p* = 0.71; χ^2^(2) = 3.67, *p* = 0.16; χ^2^(2) = 1.37, *p* = 0.51.

##### Awareness at baseline per condition

There was homogeneity of variances, as assessed by Levene’s test for equality of variances (based on mean *p* = 0.20). Awareness was not statistically significantly different between conditions, F(2, 234) = 0.77, *p* = 0.46.

##### Perceived susceptibility at baseline per condition and per distance level

There was homogeneity of variances, as assessed by Levene’s test for equality of variances (based on mean, patients abroad: *p* = 0.91; ‘your patients’: *p* = 0.65, and ‘you’: *p* = 0.75). Susceptibility was not statistically significantly different between conditions at any distance level, patients abroad: F(2, 234) = 1.49, *p* = 0.228, ‘your patients’: F(2, 234) = 2.57, *p* = 0.08, and ‘you’: F(2, 234) = 0.04, *p* = 0.96.

The difference in perceived susceptibility for the different distance levels was statistically significant, *F*(1.73, 407.64) = 114.43, *p* < 0.001, applying ε = 0.86. Post-hoc comparisons showed that perceived susceptibility was statistically significantly different between ‘patients abroad’ and ‘you’ (respectively *M* = 5.75 and *M* = 4.91 mean difference 0.84, 95% CI 0.66–1.01, *p* < 0.001) and ‘your patients’ and ‘you’ (respectively *M* = 5.67 and *M* = 4.91, mean difference 0.76, 95% CI 0.63–0.88, *p* < 0.001).

### Manipulation check: participant evaluation

#### Vividly imagining sending and receiving the message

The assumption of homogeneity of variances was violated for vividly imagining sending the message as assessed by Levene’s test for equality of variances (based on mean *p* = 0.07). Vivildy imaginging sending the message was statistically significantly different between conditions, Welch’s *F*(2, 155.73) =4.280, *p* = 0.02. Post-hoc pairwise comparisons showed that vividly imagining sending the message was only statistically significantly different between the conditions reading and control (respectively *M* = 3.86 and *M* = 4.55, mean difference −0.69, *p* = 0.01).

There was homogeneity of variances for vividly imagining receiving the message, as assessed by Levene’s test for equality of variances (based on mean *p* = 0.28). Vivildy imaginging receiving the message was not statistically significantly different between conditions, *F*(2, 234) = 2.66, *p* = 0.07.

#### Vividly imaging the situation and likelihood of experiencing

There was homogeneity of variances for vividly imagining the situation, and likelihood of experience as assessed by Levene’s test for equality of variances (based on mean respectively *p* = 0.54, *p* = 0.48). Vividly imagining the situation was statistically significantly different between the conditions reading and writing, respectively *M* = 5.49 and *M* = 5.04, *F*(1,157) = 6.11, *p* = 0.02. The item vividly imagining the situation was used as the measure of imaginability in the mediation analysis. The likelihood of experiencing was not statistically significantly different between the conditions reading and writing, *F*(1,157) = 3.22, *p* = 0.08.

#### Cause-effect relationship

The assumption of homogeneity of variances was violated for the cause-effect relationship as assessed by Levene’s test for equality of variances (based on mean *p* < 0.001). Recognizing the cause-effect relationship was statistically significantly different between the conditions reading and writing, respectively *M* = 4.63 and *M* = 5.21, Welch’s *F*(1, 138.68) = 11.12, *p* = 0.001.
